# Mutations in the macrodomain 1 of nsp3 attenuate PEDV virulence *in vivo* without impairing *in vitro* replication

**DOI:** 10.1128/jvi.01251-26

**Published:** 2026-09-09

**Authors:** Mingde Liu, Men Thi Hoai Duong, Thamonpan Laocharoensuk, Bikash Aryal, Anthony R. Fehr, Lari Lehtiö, Qiuhong Wang

**Affiliations:** 1 Center for Food Animal Health, Department of Animal Sciences, College of Food, Agricultural and Environmental Sciences, The Ohio State University 621311, Wooster, Ohio, USA; 2 Department of Veterinary Preventive Medicine, College of Veterinary Medicine, The Ohio State University 70728, Columbus, Ohio, USA; 3 Faculty of Biochemistry and Molecular Medicine & Biocenter Oulu, University of Oulu 200155, Oulu, Finland; 4 Department of Molecular Biosciences, The University of Kansas 4202, Lawrence, Kansas, USA; Loyola University Chicago - Health Sciences Campus, Maywood, Illinois, USA

**Keywords:** coronavirus, porcine epidemic diarrhea virus, PEDV, nonstructural protein 3, macrodomain, Mac1, ADP-ribosylation, ADP-ribosylhydrolase, interferon, IFN

## Abstract

The conserved macrodomain 1 (Mac1) within coronavirus nsp3 reverses ADP-ribosylation of proteins and contributes to viral virulence. While Mac1 has been characterized in several coronaviruses, its role in porcine epidemic diarrhea virus (PEDV) remains unknown. Since D445 and N462 are functionally important residues in several betacoronaviruses, we mutated the two residues in both the PEDV Mac1 recombinant protein and the infectious virus and analyzed them *in vitro* and *in vivo*. Sequence alignment revealed that N462 is highly conserved, whereas D445 is less conserved among porcine coronaviruses. The N462A mutation significantly impaired both ADP-ribose binding and hydrolysis activity of Mac1, whereas D445A had minimal effect. Compared with wild-type PEDV, both PEDV mutants (icPC22A-nsp3-D445A and icPC22A-nsp3-N462A) replicated efficiently as wild type in Vero, porcine kidney LLC-PK1, and porcine intestinal epithelial IPEC-J2 cells. Unexpectedly, both mutated viruses induced significantly lower levels of interferons in LLC-PK1 cells and IPEC-J2 cells, in contrast to previously characterized Mac1 mutants of SARS-CoV-2 and MHV-JHMV strain. Moreover, the icPC22A-nsp3-N462A virus showed significantly reduced sensitivity to IFN-β treatment in Vero cells. These results suggest that the PEDV Mac1 functions as an interferon inducer. In gnotobiotic neonatal piglets, icPC22A-nsp3-N462A showed reduced and delayed peak viral shedding and attenuated phenotype compared with wild-type PEDV. Collectively, this work provides the first *in vitro* and *in vivo* evidence that the conserved N462 residue in the PEDV Mac1 domain is critical for both ADP-ribose binding and hydrolysis, induces IFN production, and contributes to virulence. However, how these pathways are connected deserves further investigation.

**IMPORTANCE:**

Coronaviruses frequently emerge from wildlife reservoirs to infect humans and livestock, causing economically devastating epidemics/pandemics marked by severe respiratory or enteric diseases. Despite advances in vaccines and therapies, the risk of diverse coronaviruses remains a global concern. PEDV is an alphacoronavirus and causes fatal diarrhea in neonatal pigs, leading to recurring outbreaks and significant economic losses in the global swine industry. The Mac1 domain antagonizes host ADP-ribosylation and impacts pathogenesis in beta-coronaviruses, such as SARS-CoV, SARS-CoV-2, and mouse hepatitis virus (MHV). However, its role in porcine or enteric coronaviruses remains undefined. Our study revealed that PEDV Mac1 mutations decreased enzymatic function and interferon response. The latter differs from respiratory SARS-CoV and neurotropic MHV, suggesting that Mac1’s functions adapt to different host or tissue environments. Our findings identify Mac1 N462 as a rational target for live attenuated vaccine design and highlight Mac1 as a potential target for antiviral therapeutics against PEDV.

## INTRODUCTION

Coronaviruses (CoVs) are enveloped positive-sense, single-stranded RNA viruses and have the largest genomes among all known RNA viruses. CoVs are classified into four genera: *Alphacoronavirus, Betacoronavirus, Gammacoronavirus,* and *Deltacoronavirus* (1). Porcine epidemic diarrhea virus (PEDV), a member of the *Alphacoronavirus* genus, infects pigs of all ages and causes acute diarrhea, vomiting, and dehydration, resulting in up to 100% mortality in neonatal piglets (2–4). In 2013, highly virulent PEDV emerged in the United States and caused massive outbreaks and substantial economic losses to the swine industry (5–7). Currently, PEDV still poses a threat to certain US pig farms, as effective vaccines remain unavailable for PEDV-naïve pigs (8, 9). Furthermore, PEDV has become the third most impactful pathogen, next to African swine fever virus (ASFV) and Porcine reproductive and respiratory syndrome virus (PRRSV), for swine in China and continues to pose a major threat to the global pork industry (10–15).

The genome of PEDV is 28 kb long and contains a 5′-cap structure and a 3′-poly (A) tail (16). It encodes 16 nonstructural proteins (nsp1–nsp16), which are generated by proteolytic processing of the polyprotein 1a/ab (pp1a/1ab), four structural proteins (spike [S], envelope [E], membrane [M], and nucleocapsid [N]), and an accessory protein ORF3. Nsp3 is the largest multi-domain protein produced by CoVs (17), and can contain 10–16 domains depending on the CoV genera, with 8 domains and 2 transmembrane regions conserved across genera according to *in silico* analysis (18).

All CoV nsp3 proteins contain a conserved macrodomain 1 (Mac1) (19). While certain CoVs, including severe acute respiratory syndrome coronavirus 2 (SARS-CoV-2), encode additional macrodomains (Mac2 and Mac3), Mac1 is the only macrodomain conserved across all CoV nsp3 proteins. Macrodomains are globular protein domains found not only in CoVs but also across a wide range of life forms, including mammals, plants, invertebrates, fungi, archaea, and bacteria. They are also found in several other families of positive-sense RNA viruses, such as alphavirus, rubella virus, and hepatitis E virus (HEV) (20–24). The broad conservation of macrodomains across diverse organisms underscores their biological importance. In CoVs, Mac1 is highly conserved and has been established as a key virulence factor. The CoV Mac1 binds ADP-ribose moieties and hydrolyzes them from target proteins, thereby removing ADP-ribose and reversing protein ADP-ribosylation (25–29).

ADP-ribosylation is a reversible post-translational modification by which ADP-ribose is covalently attached to a target protein or nucleic acid (30, 31). ADP-ribosylation is catalyzed by PARP family enzymes known as ADP-ribosyltransferases (ARTs), including mono-ADPr-transferases (mono-ARTs) and poly-ADPr-transferases (poly-ARTs) in eukaryotic cells (32). Many mono-ARTs can be induced by interferon (IFN) to modulate the inflammatory response and to suppress viral replication, implicating the role of ADP-ribosylation in host antiviral response (33). These findings suggest that viral Mac1 antagonizes mono-ART-mediated host antiviral responses, thereby supporting viral infection, which has been demonstrated for MHV, SARS-CoV-2, Sindbis virus, and Semliki Forest Virus (34–38).

Previous studies have demonstrated that the conserved aspartic acid (D) and asparagine (N) residues within the Mac1 domain are critical for ADP-ribose binding and hydrolysis, respectively. Their mutation can significantly impact CoV replication and pathogenesis. The highly conserved asparagine has been studied for multiple CoVs, including the alpha-CoV human CoV 229E (HCoV-229E and N1305A) (27), and several beta-CoVs such as mouse hepatitis virus (MHV)-JHMV strain (N1347A), MHV-A59 strain (N1348A) (34, 36, 39–41), severe acute respiratory syndrome coronavirus (SARS-CoV) (N1040A) (27, 42), and SARS-CoV-2 (N1062A/D) (43, 44). Structural studies suggest that this N residue forms critical hydrogen bonds with the distal ribose of ADP-ribose. Previous characterizations of N-to-A or N-to-D mutations of recombinant SARS-CoV-2 Mac1 protein disrupted the hydrolysis activity but did not affect the ADP-ribose binding capability (43, 45). The N-to-A mutations generally do not significantly impair viral replication in cell lines, with the exception of the MHV-JHMV mutant (N1347A), which replicated poorly in primary bone marrow-derived macrophages (BMDMs) (34, 41). However, these mutants induced elevated IFN responses and were highly attenuated *in vivo* (27, 39, 40, 42, 43).

In contrast, D-to-A mutations in SARS-CoV-2 and Middle East respiratory syndrome coronavirus (MERS-CoV) Mac1 proteins did not impair hydrolysis activity but drastically reduced ADP-ribose binding (41). In SARS-CoV and SARS-CoV-2, the D-to-A mutant replicated normally in cell culture but was attenuated in mice (42, 44). However, the corresponding MHV-JHMV D1329A mutant was highly attenuated in all tested cell types, much more than the N1347A mutant in the same MHV-JHMV strain (39, 41, 46). Moreover, an MHV-JHMV mutant carrying both D1329A and N1347A substitutions could not be rescued, while SARS-CoV-2 mutant with the whole Mac1 deleted was viable, suggesting that ADP-ribosylhydrolase activity may have different roles during CoV infection depending on the virus and cell type.

Despite many studies on Mac1 in several CoVs, its biological role remains elusive in porcine and enteric CoVs. PEDV infects pigs and primarily targets the small intestine (7), whose major tropism is different from previously studied CoVs. Studying PEDV Mac1 may therefore help uncover how this domain contributes to viral replication, immune evasion, and virus-host interactions in the context of enteric infection and a distinct host species.

In this study, we evaluated the role of the D445 and N462 residues in PEDV Mac1 function by generating individual alanine substitutions into the Mac1 recombinant proteins and the infectious viruses and characterized their effects *in vitro* and *in vivo*. Our results demonstrated that the conserved N462 residue is essential for ADP-ribose binding and hydrolysis activity, and that its substitution attenuates viral virulence. These findings provide the first functional characterization of porcine and enteric Mac1 and highlight its potential as a novel attenuation target for PEDV live-attenuated vaccine development.

## RESULTS

### The D445 and N462 residues are conserved in the Mac1 domain of PEDV nsp3

**Fig 1:**
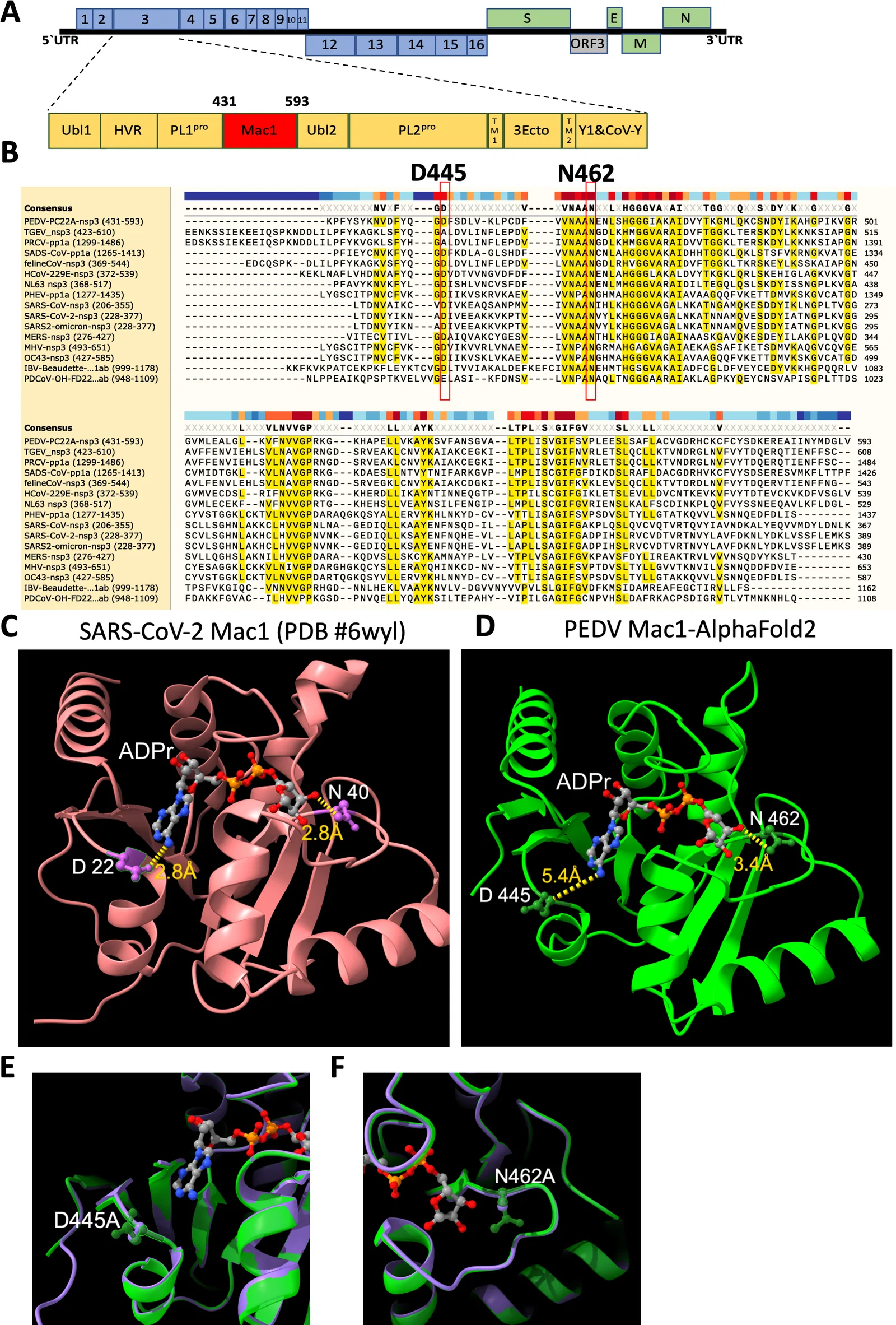
Conservation and structural modeling of the nsp3 Mac1 domain of coronaviruses. (**A**) Schematic diagram of the genomic organization of PEDV and the location of the nsp3 Mac1 domain. Nsp3 domains are presented based on the HCoV NL63 strain (17, 47). (**B**) Sequence alignment of the Mac1 domains of representative coronaviruses (PRCV, porcine respiratory coronavirus; SADS-CoV, swine acute diarrhea syndrome coronavirus; PHEV, porcine hemagglutinating encephalomyelitis virus; OC43, human coronavirus OC43). Amino acid residues D445 and N462 are boxed. (**C**) Crystal structure of SARS-CoV-2 Mac1 domain in complex with ADP-ribose (PDB: 6ywl) (48). Key residues D22 and N40 are shown as ball-and-stick models with darker color. In both panels C and D, ADP-ribose (ADPr) is displayed in ball-and-stick model. (**D**) AlphaFold2-predicted structure of PEDV Mac1 with ADP-ribose ligand added using AlphaFill. Key residues D445 and N462 are shown as ball-and-stick models with darker color. The distances between the key residues and the ligand are indicated by yellow dashed lines and labeled in yellow (unit: Å). Zoomed-in figure of the PEDV Mac1 at the D445A (**E**) and N462A (**F**) mutation sites. Wild-type residues are shown as a ball-and-stick model (lime), and the alanine mutations are shown in purple.

The Mac1 domain of PEDV encompasses amino acid residues 431–593 of nsp3 based on sequence alignment and 3D structure prediction (Fig. 1). Sequence alignment of the Mac1 domains of representative alpha-, beta-, gamma-, and delta-CoVs demonstrated a high level of conservation of core residues (Fig. 1B). Among them, the aspartic acid (D445, conserved in most CoVs) and asparagine (N462, conserved in all CoVs) residues have been well characterized in beta-CoVs. Intriguingly, the D residue is less conserved than the N residue among CoVs, especially in porcine CoVs (Fig. 1B). Porcine enteric CoV transmissible gastroenteritis virus (TGEV) and porcine respiratory CoV (PRCV) have alanine (A) and porcine deltacoronavirus (PDCoV) has glutamic acid (E) at their corresponding residues of PEDV D445, suggesting that other residues may compensate for ADP-ribose binding.

Like previously characterized CoV Mac1 domains (HCoV-229E, HCoV-NL63, SARS-CoV, MERS-CoV, SARS-CoV-2, and infectious bronchitis virus [IBV]) (26, 49–54), PEDV Mac1 features a similar three-layered α/β/α sandwich structure, with a central β sheet composed of seven β strands, flanked by three α-helices on each side. Structural alignment of the AlphaFold2 predicted PEDV Mac1 and the published crystal structure of SARS-CoV-2 Mac1 domains (PDB #6ywl) (48) revealed a highly similar overall fold, with an RMSD of 0.93 Å across 125 Cα atoms and ADP-ribose positioned in a conserved binding pocket (Fig. 1C and D). Interestingly, in the SARS-CoV-2 Mac1 (PDB #6ywl), both D22 and N40 lay within 2.8 Å of the ADP-ribose, suggesting direct interaction (Fig. 1C). However, AlphaFold2 predicted PEDV Mac1 3D structure (Fig. 1D) revealed residue N462 positioned 3.4 Å from ADP-ribose, while D445 is predicted to be at 5.4 Å distance from ADP-ribose, which is nearly twice the distance of SARS-CoV-2 corresponding D22. This suggests a weaker interaction between PEDV Mac1 D445 residue and ADP-ribose, providing a possible explanation for the reduced conservation of this D445 residue among porcine CoVs and raising the question of whether D445 plays the same functional role in PEDV as it does in other CoVs.

Predicted structural models of PEDV Mac1 D445A (Fig. 1E) and N462A (Fig. 1F) mutants were created to visualize the mutation-associated potential changes at these sites. The models illustrate localized alterations in side chain conformations without causing major changes to the overall fold of the Mac1 domain.

### ADP-ribose binding and ADP-ribosyl hydrolase activities of recombinant PEDV Mac1 protein and its mutants

**Fig 2:**
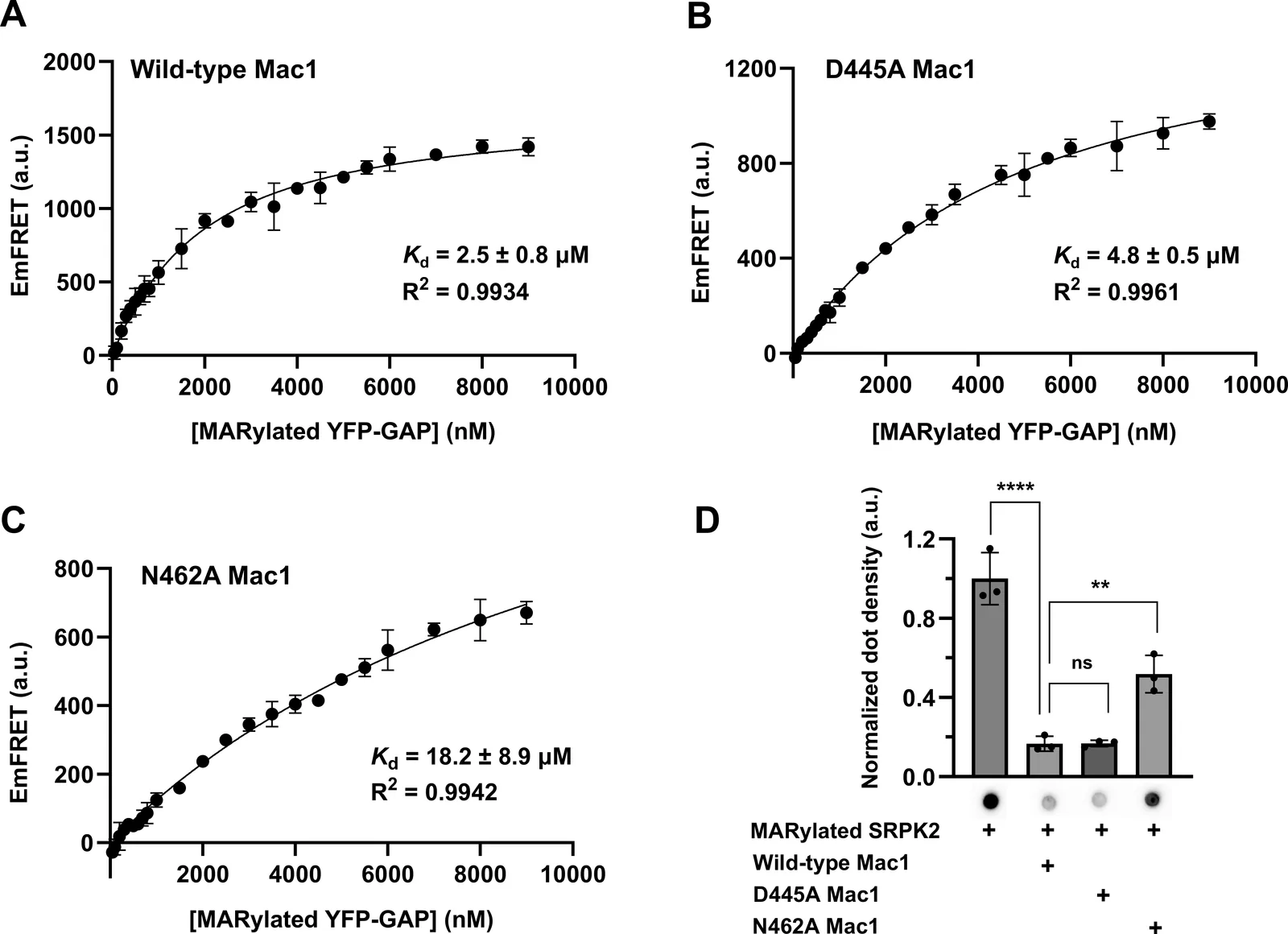
Comparison of the ADP-ribosyl binding affinity and hydrolysis activity between wild-type PEDV Mac1 and two single-point mutants (D445A Mac1 and N462A Mac1). Determination of dissociation constant (*K*_d_) between CFP–wild-type Mac1 (**A**), CFP–D445A Mac1 (**B**), CFP–N462A Mac1 (**C**), and MARylated YFP-GAP using a FRET-based method (55). Data are shown as mean ± standard deviation with four replicates. *K*_d_ values are shown as mean ± standard deviation from three independent experiments. (**D**) The hydrolysis activities of wild-type Mac1 and Mac1 mutants were measured using a dot blot method with MARylated SRPK2 as the substrate. Representative dots are shown. Data are displayed as mean ± standard deviation with *n* = 3. Individual data points are shown (****, *P* < 0.0001; **, *P* < 0.005; ns, not significant).

To evaluate the roles of residues D445 and N462 in the biochemical function of PEDV Mac1 *in vitro*, we expressed recombinant wild-type PEDV Mac1 protein (K431–V593) and its single-point mutants, D445A Mac1 and N462A Mac1. To assess the ADP-ribosyl binding activity of Mac1 to an ADP-ribosylated protein, we employed a fluorescence resonance energy transfer (FRET)-based method and determined the dissociation constant (*K*_d_) between Mac1 and a mono-ADP-ribosylated Yellow Fluorescent Protein–Gi protein alpha subunit peptide (MARylated YFP-GAP) (55, 56). Wild-type Mac1 exhibited a strong binding affinity to MARylated GAP with a *K*_d_ value of 2.5 ± 0.8 µM, while the D445A mutation resulted in a slight reduction in binding affinity (*K*_d_ = 4.8 ± 0.5 µM), and the N462A mutation even further impaired the binding (*K*_d_ = 18.2 ± 8.9 µM) (Fig. 2A through C). These results are consistent with structural alignment data and suggest that D445 is positioned distally and might have weaker interactions with the ADP-ribose, while N462 is in close contact with the distal ribose moiety and likely contributes more critically to binding.

To further evaluate whether those mutations affect the hydrolysis activity of PEDV Mac1, we performed a dot blot assay using MARylated SRPK2 protein as the substrate. The ADP-ribosylated protein blotted on the membrane was detected using a luminescent nanoluciferase-fused ADP-ribosyl superbinder eAf1521 (56). The D445A mutation did not alter the ADP-ribosyl hydrolase activity of Mac1, which may be expected based on its limited impact on ADP-ribose binding and prior results in other CoVs that have shown that this D-A mutation generally retains some enzyme activity. In contrast, the N462A mutation significantly reduced the enzymatic activity, resulting in an approximately 50% decrease in the hydrolysis efficiency of Mac1 (Fig. 2D). Altogether, these results demonstrate that while D445 might not be essential to maintain the biochemical function of Mac1 *in vitro*, N462 plays a critical role, and its mutation to alanine significantly impairs both the ADP-ribosyl binding and hydrolysis activities of PEDV Mac1 *in vitro*.

### PEDV Mac1-mutant viruses were rescued and characterized in cell culture

**Fig 3:**
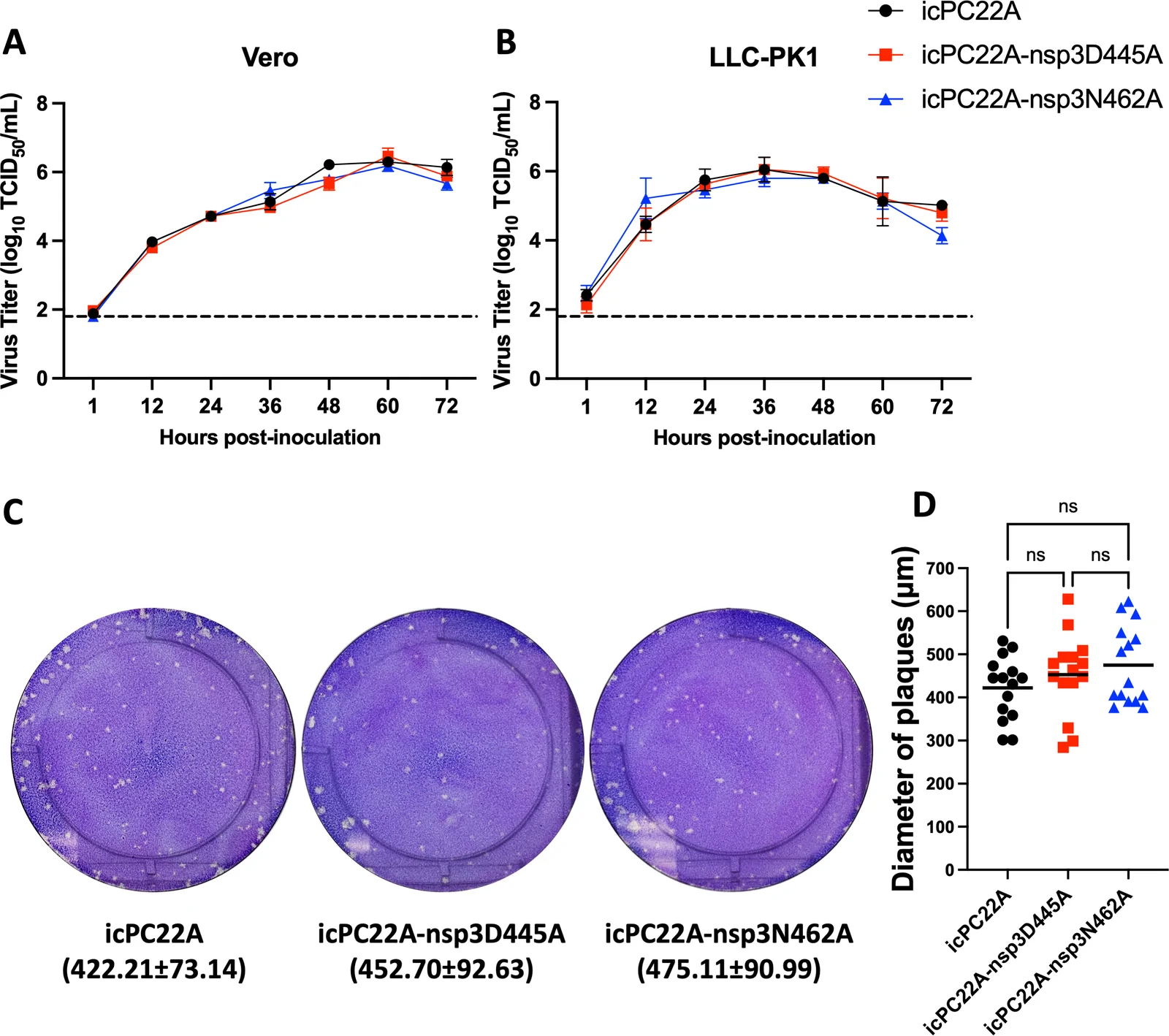
Growth kinetics and plaques of wild-type and PEDV Mac1 mutants in cell cultures. Multistep growth kinetics of wild-type PEDV icPC22A, and two Mac1 mutants (icPC22A-nsp3D445A and icPC22A-nsp3N462A) in Vero (**A**) and LLC-PK1 (**B**) cells infected with each virus at a multiplicity of infection (MOI) of 0.01. Viral infectious titers were determined in Vero cells as TCID_50_/mL. Limit of detection (1.8 log_10_ TCID_50_/mL) was indicated with a dash line. (**C**) Representative plaque morphologies of each virus on Vero cells. (**D**) The diameters of 15 plaques were measured and plotted, and the mean was shown as a line. ns, not significant.

To further characterize the roles of residues D445 and N462 in PEDV Mac1 during viral replication, we introduced alanine substitutions at either D445 or N462 in the PEDV infectious clone icPC22A via reverse genetics (57). The recombinant viruses icPC22A-nsp3D445A and icPC22A-nsp3N462A were rescued in Vero cells. The complete genome sequences of the virus stocks used for the following experiments were verified. We compared the multistep growth kinetics of wild-type PEDV icPC22A and the two mutants in Vero and LLC-PK1 cells at a multiplicity of infection (MOI) of 0.01. In both Vero (Fig. 3A) and LLC-PK1 cells (Fig. 3B), all three viruses displayed similar replication kinetics, reaching similar peak titers at similar hours post-infection (hpi). Plaque assays were performed on Vero cells, and the plaques from each virus showed similar size and morphology (Fig. 3C and D).

### Induction of type I and type III interferon responses by PEDV Mac1 mutants in cell culture

**Fig 4:**
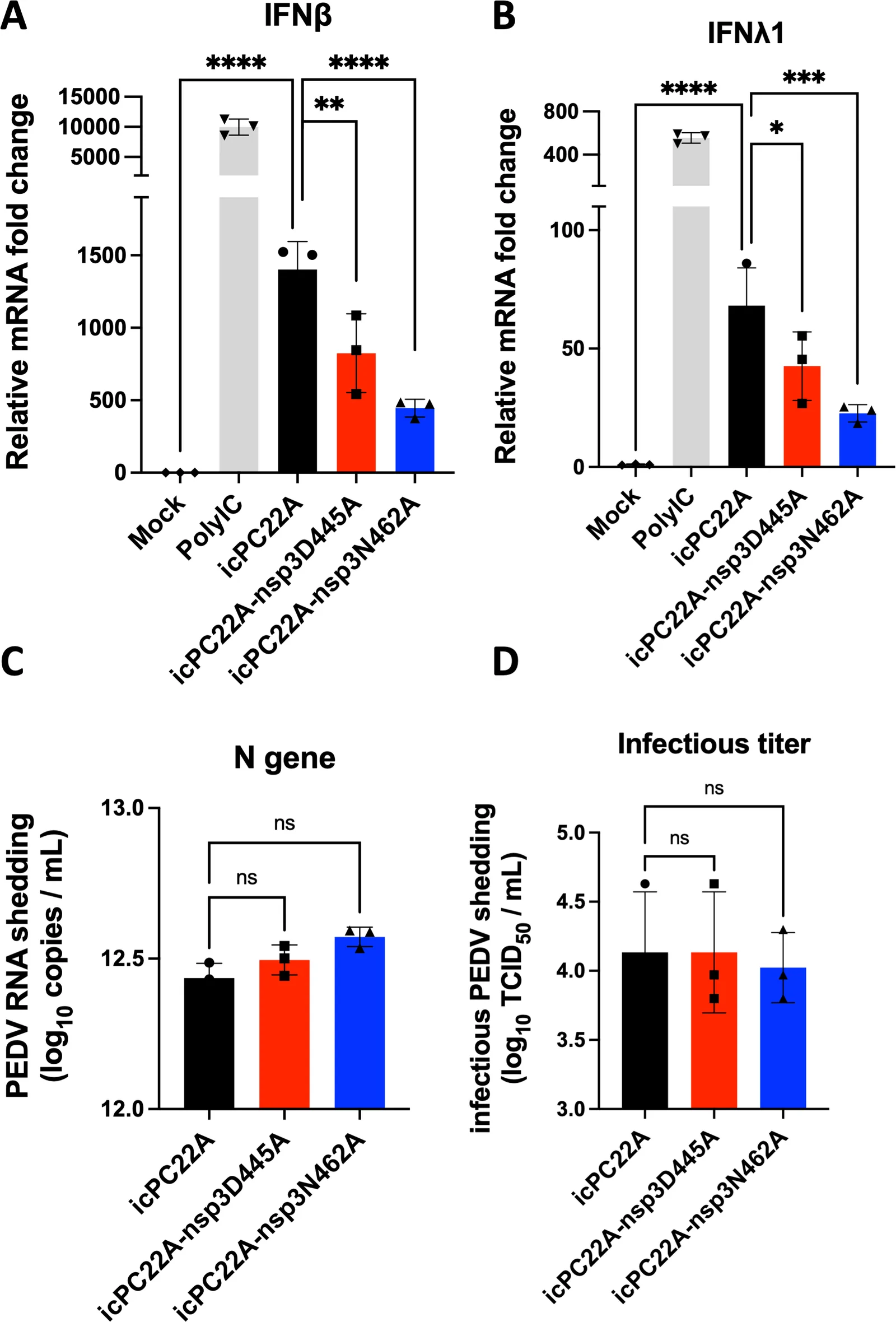
Induction of type I and type III IFN mRNAs by wild-type PEDV and the PEDV mutants in LLC-PK1 cells. The cells were inoculated with the indicated virus at an MOI of 1 or medium (mock) as a negative control or stimulated with poly I:C as a positive control. At 12 hpi, cell monolayers were lysed for measuring mRNA levels of IFN-β (**A**), IFN-𝜆1 (**B**), and the PEDV total N gene (**C**), and the supernatants were collected for testing viral infectious titers in TCID_50_ (**D**). Groups with significant differences from icPC22A were indicated. Data are displayed as mean ± standard deviation (*n* = 3) from one experiment representative of three independent trials. Individual data points are shown (*, *P* < 0.05; **, *P* < 0.01; ***, *P* < 0.005; ****, *P* < 0.001; and ns, not significant).

Because Vero cells are IFN-deficient, we investigated whether the Mac1 mutations altered IFN induction during infection of LLC-PK1 cells by RT-qPCR. Compared with wild-type PEDV, both Mac1 mutants induced significantly lower levels of IFN-β (Fig. 4A) and IFN-λ1 (Fig. 4B) transcripts in porcine kidney LLC-PK1 cells at 12 hpi, with a higher degree for icPC22A-nsp3N462A than icPC22A-nsp3D445A. No significant differences in viral N gene RNA levels (Fig. 4C) and viral infectious titers (Fig. 4D) were found among the three viruses, suggesting that the reduced IFN induction was not due to differences in viral loads at the time of sampling.

### Differential gene expression (DGE) analysis of IPEC-J2 cells infected with individual nsp3 mutants

**Fig 5:**
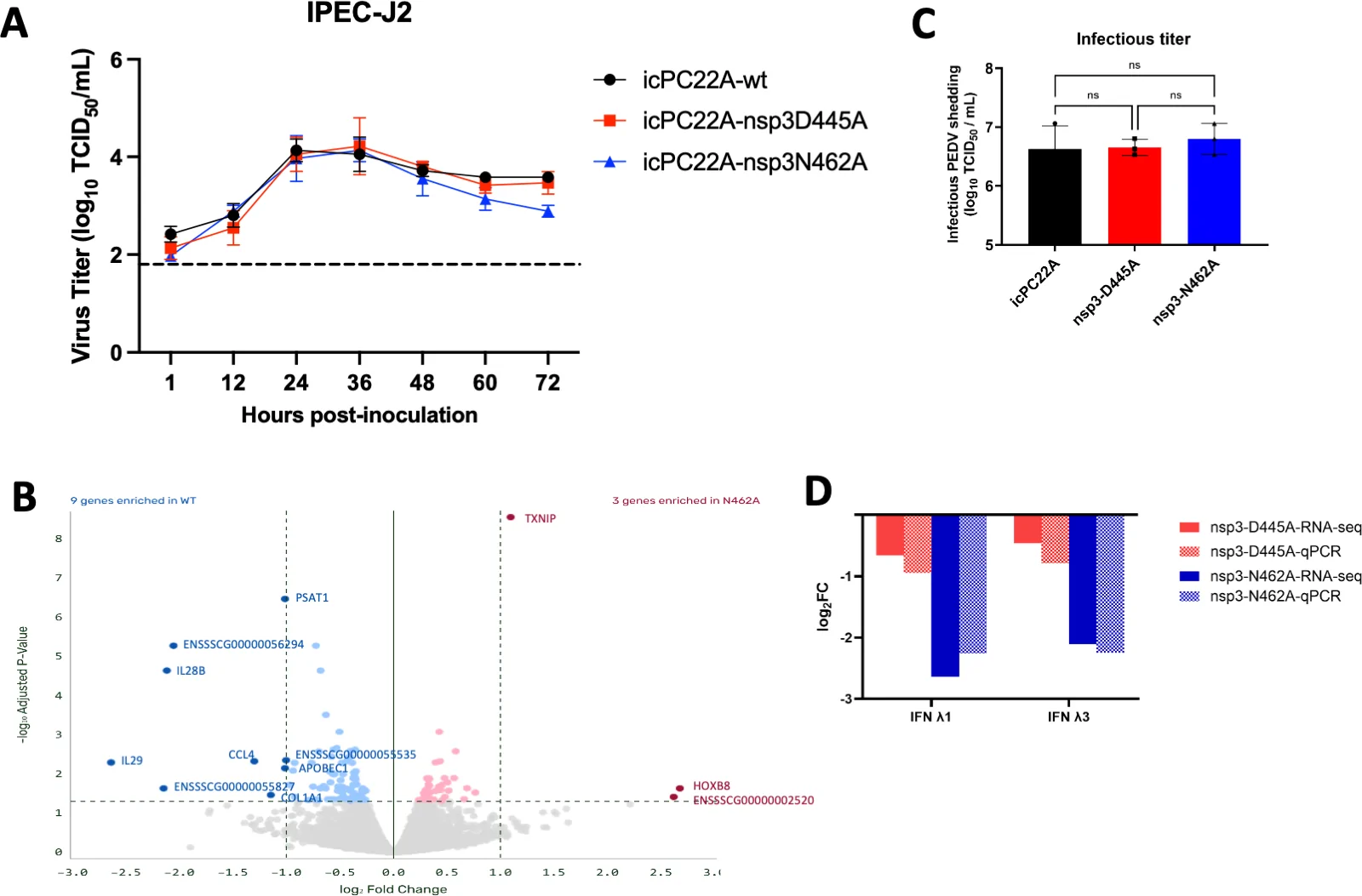
Multistep growth kinetics and RNA-seq analyses of virus-infected IPEC-J2 cells. (**A**) Multistep growth kinetics of wild-type PEDV icPC22A and two Mac1 mutants, icPC22A-nsp3D445A and icPC22A-nsp3N462A, in IPEC-J2 cells infected with each virus at a multiplicity of infection (MOI) of 0.01. Viral infectious titers were determined at the indicated time points and shown in TCID_50_/mL. Limit of detection (1.8 TCID_50_/mL) was indicated with a dashed line. (**B–D**) The cells were inoculated with the indicated virus at an MOI of 3. At 12 hpi, the cells were lysed for RNA extraction for RNA-seq analyses (**B**) and for RT-qPCR for IFN λ1 and IFN λ3 mRNA levels (**D**), and the supernatants were collected for testing viral infectious titers in TCID_50_ (**C**). (**B**) Differentially expressed genes (DEGs) of IPEC-J2 cells infected with icPC22A-nsp3N462A mutant. Volcano plot of DEGs in the icPC22A-nsp3N462A-infected IPEC-J2 cells. The *x*-axis represents the log2 transformation of gene expression level relative to the icPC22A-infected IPEC-J2 cells, while the *y*-axis shows the log10 transformed adjusted *P* value. Vertical dashed lines indicate the cutoff for log2 fold change (|log2FC| ≥ 1), and the horizontal dashed line indicates the adjusted *P* value cutoff (adj-*P* < 0.05). Gene symbols or Ensembl gene IDs for the significant DEGs are shown. Red dots and blue dots indicate upregulated and downregulated genes in the nsp3N462A-infected group, respectively. (**C**) Infectious viral titers from supernatants were determined in TCID_50_. Groups with not significant (ns) differences compared with icPC22A were indicated. (**D**) Validation of selected DEGs, IFN λ1 (IL29) and IFN λ3 (IL28B), by RT-qPCR to validate the RNA-seq findings.

To evaluate whether the immunomodulatory effects of Mac1 mutations are cell-type specific, we further examined viral replication and cellular responses in the porcine intestinal epithelial cell line IPEC-J2, which more closely reflects the natural tropism of PEDV. Wild-type icPC22A and Mac1 mutant viruses (icPC22A-nsp3D445A and icPC22A-nsp3N462A) replicated with similar multistep growth kinetics in IPEC-J2 cells (Fig. 5A). To gain further insight into the IFN responses induced by the Mac1 mutants and other downstream cellular pathways affected, we investigated DEGs in cells infected with each individual nsp3 mutant (MOI = 3) compared with wild-type PEDV icPC22A (icPC22A vs icPC22A-nsp3D445A and icPC22A vs icPC22A-nsp3N462A) using RNA sequencing (RNA-seq). There were no significant DEGs between the icPC22A and icPC22A-nsp3D445A groups. In contrast, 12 significant DEGs were identified between the icPC22A and icPC22A-nsp3N462A groups (Fig. 5B), of which 3 genes (ENSSSCG00000002520, HOXB8, and TXNIP) were upregulated and 9 genes (APOBEC1, CCL4, COL1A1, ENSSSCG00000055535, ENSSSCG00000055827, ENSSSCG00000056294, IL28B or IFN λ3, IL29 or IFN λ1, and PSAT1) were downregulated in icPC22A-nsp3N462A-infected cells. The identified DEGs were unlikely to result from differences in viral load, as infectious viral titers were not significantly different among the three groups (Fig. 5C). Among the significant DEGs, several genes, including CCL4, COL1A, IL28B, IL29, and TXNIP, have been reported to be associated with ADP-ribosylation-related pathways (Table S1) (38, 58–62). For example, in primary macrophages, TXNIP (thioredoxin-interacting protein) interacts with PARP1 and regulates NLRP3 inflammasome activation through PARylation (58). Functional enrichment analysis of downregulated genes revealed enrichment of pathways related to vitamin B6 metabolism (ssc00750; gene: PSAT1), JAK-STAT signaling pathway (ssc04630; genes: ENSSSCG00000056294, IL28B, and IL29), and cytokine-cytokine receptor interaction (ssc04060; CCL4, ENSSSCG00000056294, IL28B, and IL29) (Table 1). To further validate DEGs from RNA-seq, RT-qPCR was performed for IFN-λ1 (IL29) and IFN-λ3 (IL28B). The relative expression of IFN-λ1 and IFN-λ3 genes was consistent with the RNA-seq results (Fig. 5D).

**TABLE 1:** Pathway enrichment analysis of downregulated DEGs in the nsp3N462A-infected IPEC-J2 cells using ShinyGO (v0.85.1)

Enrichment false discovery rate	nGenes	Pathway genes	Fold enrichment	Pathway	Genes
0.022843	1	8	393.625	Path:ssc00750 Vitamin B6 metabolism	PSAT1
0.00017	3	159	59.41509	Path:ssc04630 JAK-STAT signaling pathway	ENSSSCG00000056294 IL29 IL28B
1.63E−05	4	256	49.20313	Path:ssc04060 Cytokine-cytokine receptor interaction	ENSSSCG00000056294 IL29 IL28B CCL4

### Sensitivity of PEDV Mac1 mutants to IFN-β pre-treatment in Vero cells

**Fig 6:**
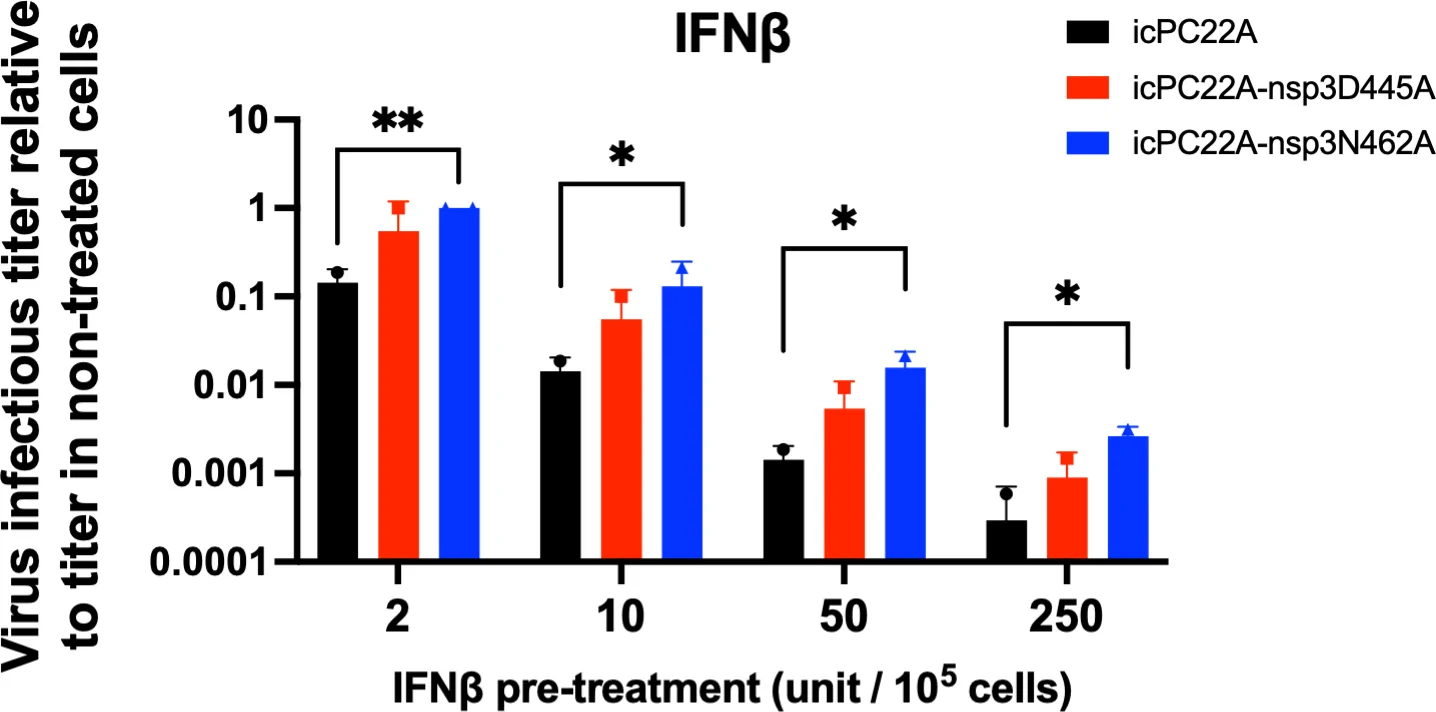
Sensitivity of the PEDV mutants to IFN-β pre-treatment in Vero cells. Cells were pre-treated with different concentrations of IFN-β for 18 h and inoculated with each virus at an MOI of 0.01. Values indicate the ratio of the viral infectious titers of the treated cells relative to those of the non-treated cells at 24 hpi. Groups with significant differences from icPC22A were indicated. Data are displayed as mean ± standard deviation (*n* = 3) from one experiment representative of three independent trials. Individual data points are shown (*, *P* < 0.05; **, *P* < 0.01).

To assess whether the Mac1 mutations altered downstream IFN responsiveness, we evaluated the sensitivity of each virus to exogenous IFN-β pre-treatment for 18 h in Vero cells, which retain IFN receptor signaling pathways and are a standard model to assess PEDV IFN-β sensitivity (63). Infectious viral titers were measured by TCID_50_ at 24 hpi and normalized to titers from non-treated cells. All three viruses showed dose-dependent inhibition of replication by IFN-β pre-treatment, but icPC22A-nsp3N462A, and not icPC22A-nsp3D445A, replicated to significantly higher titers than wild-type PEDV across all tested concentrations (Fig. 6). These findings are consistent with the results of decreased type I and type III IFN responses induced by PEDV Mac1 mutants in LLC-PK1 cells, suggesting that PEDV Mac1 both enhanced IFN production and made PEDV more vulnerable to IFN-triggered antiviral responses. However, these results are different from the Mac1 D-to-A and N-to-A mutants of other previously studied CoVs (39, 40, 43, 45, 46).

### *In vivo* evaluation of the PEDV icPC22A-nsp3N462A mutant in gnotobiotic (Gn) neonatal piglets

**Fig 7:**
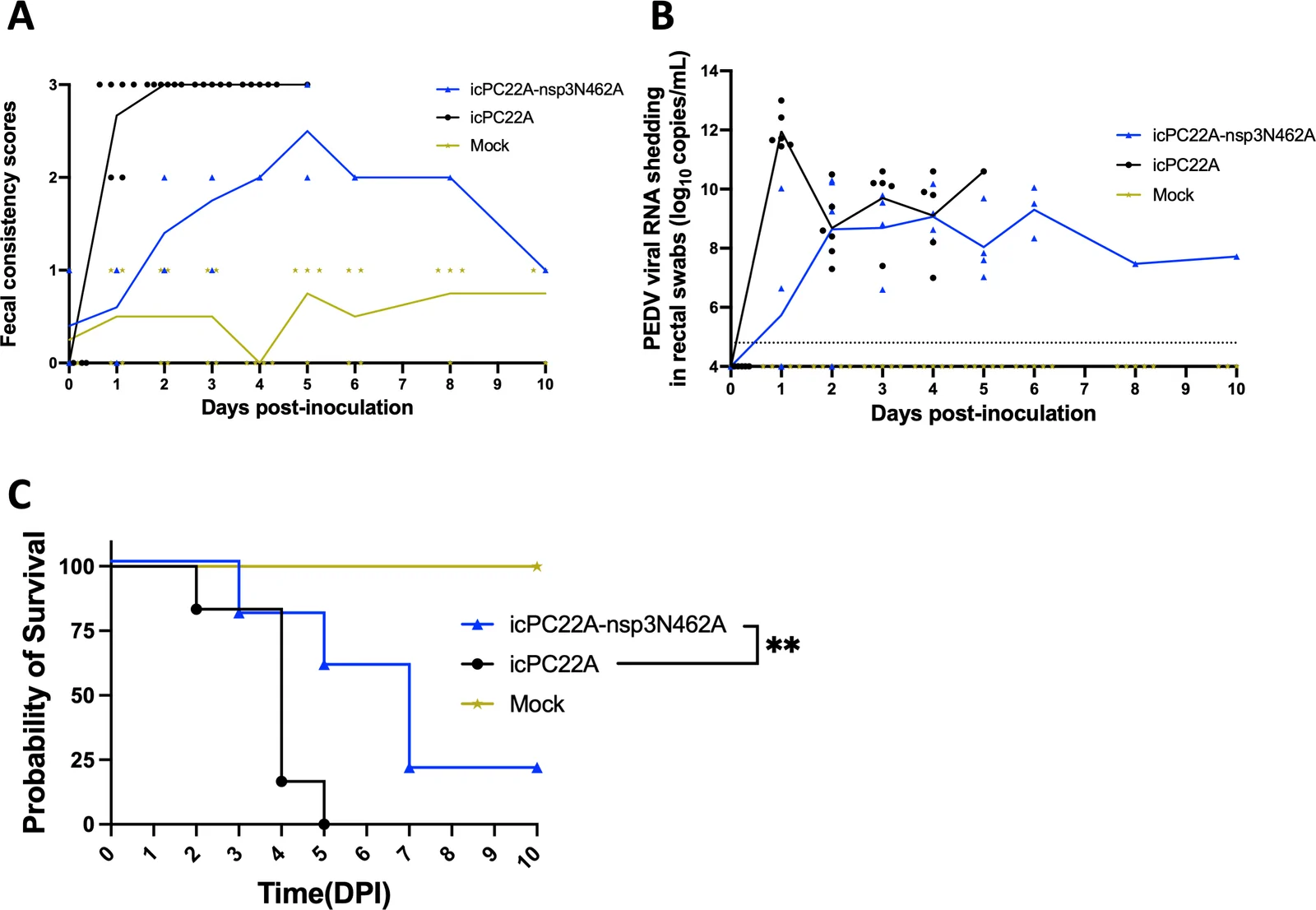
Evaluation of the replication and pathogenesis of the PEDV mutant icPC22A-nsp3N462A in 3-day-old Gn piglets. Mock group *n* = 5, icPC22A group *n* = 6, and icPC22A-nsp3N462A group *n* = 5. Each line represents the mean value of each group and the values of individual Gn piglets are also shown. (**A**) Fecal consistency scores (0, solid; 1, pasty; 2, semiliquid; and 3, liquid). A score of ≥ 2 was considered diarrhea. (**B**) Fecal PEDV RNA shedding profile. The dashed line at 4.80 log_10_ copies/mL indicates the detection limit. (**C**) Survival curves of groups. Survival differences were analyzed using the Log-rank (Mantel-Cox) test (**, *P* < 0.01).

Structural modeling predicted that N462 was positioned closer to ADP-ribose than D445 (Fig. 1D). A biochemical assay proved that the N462A mutation significantly impaired both the ADP-ribosyl binding and hydrolysis activities of the Mac1 protein, while D445A did not (Fig. 2). Between the two Mac1 mutants, the N462A mutant consistently showed significant changes in type I and type III IFN responses in LLC-PK1 cells (Fig. 4A and B) and IFN-β sensitivity in Vero cells (Fig. 6). Therefore, we assessed the replication and pathogenicity of icPC22A-nsp3N462A in 3-day-old Gn piglets. The piglets were orally inoculated with culture medium (as mock, *n* = 5), icPC22A (as positive control, *n* = 6), or icPC22A-nsp3N462A (*n* = 5), and monitored for clinical signs, viral shedding, and survival for 10 days. No pigs in the mock group developed diarrhea. Both PEDV-inoculated groups developed diarrhea, but the icPC22A group showed earlier onset and more severe diarrhea compared with the icPC22A-nsp3N462A group (Fig. 7A). Fecal viral RNA shedding, measured by RT-qPCR, showed that icPC22A-nsp3N462A reached lower peak titers and at later time points compared with the icPC22A, indicating its slower and less efficient replication *in vivo*, consistent with the fecal consistency scores (Fig. 7B). Survival analysis revealed improved outcomes in piglets inoculated with the icPC22A-nsp3N462A mutant, with delayed or reduced mortality rate relative to the wild-type virus group (Fig. 7C). The mock-inoculated group showed no mortality.

### Histopathological examination

**Fig 8:**
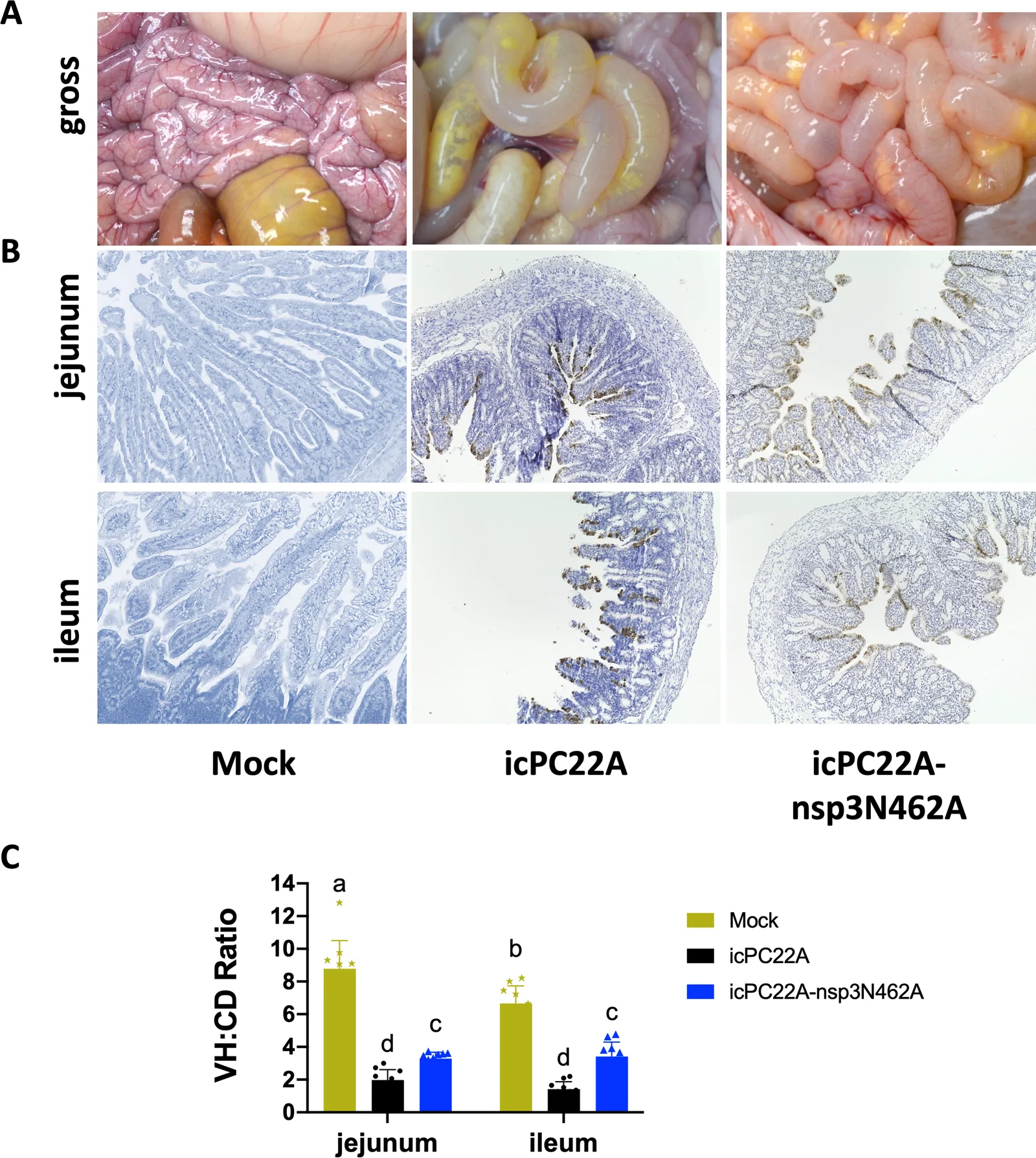
Histopathological examination of the small intestines of Gn piglets euthanized at two dpi. (**A**) Gross image of pig intestines and (**B**) immunohistochemical staining of PEDV N proteins (brown signals) in the jejunum and ileum sections of the piglets (magnification, ×100). (**C**) Villous height to crypt depth (VH:CD) ratios of piglets. Ten villi of each intestinal section were measured. Groups with significant differences (*P* < 0.05) were indicated with different letters. The error bars indicate standard deviations.

To assess the extent of small intestinal damage caused by the icPC22A-nsp3N462A mutant *in vivo*, we performed gross and histopathological examination. As expected, piglets infected with the highly virulent wild-type strain (icPC22A) displayed classic signs of severe enteric disease, including thin and transparent intestines filled with yellow watery fluid. In contrast, piglets infected with the icPC22A-nsp3N462A mutant showed slightly less intestinal distension and less transparent intestine wall, suggesting reduced fluid accumulation and tissue damage (Fig. 8A). Histological analysis further confirmed the differences in pathogenesis. In both the jejunum and ileum, icPC22A-infected piglets exhibited marked villus atrophy, blunting, and epithelial shedding, consistent with more severe PEDV-induced enteritis. However, the icPC22A-nsp3N462A mutant–infected pigs displayed moderately shortened villi with partially preserved villus architecture, indicating partial attenuation. Mock-infected controls retained normal villus morphology. Immunohistochemical (IHC) staining for PEDV N proteins showed reduced viral antigens in the intestines of icPC22A-nsp3N462A-infected piglets compared with those infected with icPC22A, correlating with the attenuated clinical phenotype (Fig. 8B). Quantitative measurement of the villus height to crypt depth (VH:CD) ratio supported these observations. icPC22A-infected piglets had significantly reduced VH:CD ratios in both the jejunum and ileum compared to mock-infected controls, while the icPC22A-nsp3N462A mutant group displayed intermediate VH:CD values that were significantly higher than the icPC22A-infected group but lower than mock (Fig. 8C). These results demonstrated that the N462A mutation in PEDV Mac1 leads to partial attenuation in neonatal piglets.

## DISCUSSION

The conserved Mac1 domain is an ADP-ribosylhydrolase and suppresses host immunity in beta-CoVs, but its function in alpha- and enteric CoVs like PEDV remains unknown. To address this knowledge gap, we targeted two conserved residues—D445 and N462—for which previous studies identified a role in ADP-ribose binding or hydrolysis function in other CoVs. The biochemical assay revealed that the D445A mutation in recombinant PEDV Mac1 protein had minimal impact on either ADP-ribosyl binding or hydrolysis activities, while the N462A mutation significantly impaired both functions. This result is different from previous findings for SARS-CoV-2 and MERS-CoV, where N-to-A mutations reduced hydrolysis activity with little impact on binding, and D-to-A mutations mainly impaired binding while retaining enzymatic function (41, 43, 45). This probably explains why this D445 residue is conserved in many CoVs but not among porcine CoVs, being an alanine (A) in TGEV and PRCV, and a glutamic acid (E) in PDCoV (Fig. 1B).

While these biochemical assays provide valuable insights into PEDV Mac1 enzymatic activity, they are performed *in vitro* using artificial substrates and may not fully represent conditions during viral infection, promoting us to further examine how these mutations affect viral replication and IFN responses in cell culture. Although neither mutation impacted virus replication *in vitro*, both, particularly N462A, significantly decreased type I and type III IFN levels in LLC-PK1 cells (Fig. 4). In addition, the N462A substitution significantly reduced the sensitivity of PEDV to IFN-β pre-treatment in Vero cells (Fig. 6). RNA-seq data of virus-infected IPEC-J2 cells revealed that the N462A mutation significantly changed 12 cellular mRNA levels, including the downregulated type III IFN (IL28B and IL29) levels. This phenotype contrasts with the reports for SARS-CoV (42) and SARS-CoV-2 (43, 44), where Mac1 mutations enhanced IFN responses by impairing ADP-ribosylhydrolase activity and allowing immune sensor activation. One possible explanation of this diverse IFN phenotype is that these differences arise from host- or tissue-specific adaptation. Interestingly, infection with Mac1-deficient MHV-JHM (a neurotropic strain carrying the N1347A) resulted in enhanced type I IFN induction in BMDMs (46), consistent with observations in SARS-CoV and SARS-CoV-2. However, in mouse brain, this Mac1-deficient MHV-JHM induced a lower level of IFN-β than wild type, though this was possibly due to reduced viral loads in the mice (39). On the other hand, infection with Mac1-deficient MHV-A59 (a hepatotropic strain carrying the N1348A) induced higher levels of IFN in primary plasmacytoid dendritic cells, but not in the spleen, liver, and sera of mice (40). These strikingly different phenotypes in different tissues suggest that whether CoV Mac1 acts as an IFN antagonist or inducer may be tissue- or virus-dependent. PEDV mainly targets porcine intestinal epithelial cells, where innate immune sensing pathways differ from non-intestinal tissues. These unexpected results suggest a divergent or even opposite immunological function of Mac1 in enteric CoVs.

PEDV Mac1 appears to induce IFN in porcine kidney LLC-PK1 cells and porcine intestinal IPEC-J2 cells. However, the mutant virus icPC22A-nsp3N462A did not replicate to higher titers *in vitro* and instead showed reduced replication and attenuated pathogenicity *in vivo*. RNA-seq analyses of virus-infected IPEC-J2 cells identified 12 significant DEGs in icPC22A-nsp3N462A-infected cells compared with wild-type icPC22A. Besides the downregulated type III IFN responses, other changed cellular pathways may counterbalance any potential replication advantage, which can be investigated in future studies. The discrepancy between the replication phenotype of icPC22A-nsp3N462A in cultured cells and in pigs further suggests that the contribution of Mac1 to viral fitness is context-dependent. The intestinal environment *in vivo* is more complex than permissive cell-culture systems and contains additional cellular, immune, and physiological pressures that may reveal functions of Mac1 that are not apparent *in vitro*. Viral proteins are shaped by selective pressures in their natural hosts, and disruption of the conserved Mac1 enzymatic activity may therefore impose a fitness cost that becomes evident primarily *in vivo*. A similar phenotype has been reported for SARS-CoV Mac1 mutants, which replicated efficiently in cultured cells but exhibited reduced replication and pathogenicity in mice (40, 42). Thus, our findings support a role for PEDV Mac1 in viral fitness and pathogenesis in the natural host, although the specific host pathways responsible for this phenotype remain to be determined.

Furthermore, an interesting question is why PEDV retains Mac1 as an IFN inducer despite encoding multiple IFN antagonists (nsp1, nsp15, nsp16, etc.) (64). We hypothesize that during enteric infections, viruses may benefit from a moderate level of local IFN responses that contribute to diarrhea, thereby enhancing viral dissemination. One example is that rotavirus-induced type III IFN (IFN-λ) signaling triggered rapid onset of diarrhea (65). Although diarrhea serves as a host defense mechanism to expel pathogens, it can paradoxically aid viral transmission by promoting rapid viral shedding and maintaining a moist environment favorable for virion stability. Thus, as an enteric virus, PEDV may benefit from maintaining IFN responses within an optimal range that permits efficient replication while also contributing to sufficient diarrhea that favors transmission. This hypothesis on enteric viruses remains speculative and deserves further investigation.

The Mac1’s impact on IFN sensitivity also differed from patterns reported in other CoVs. For example, Mac1-deficient MHV-JHMV, SARS-CoV (HKU-39849 strain), and HCoV-229E mutants were more sensitive to type I IFNs (34, 66), while Mac1 deletion or mutation in SARS-CoV-2 increased sensitivity to IFN-γ but not IFN-β (43, 44). Studies on MHV-A59 and SARS-CoV-MA15 reported no significant increase in IFN sensitivity upon Mac1 inactivation (39, 40, 42). These varied outcomes suggest that the impact of Mac1 on IFN sensitivity is influenced by both viral and host factors, such as viral genetic background, IFN subtype, and host cell type. Notably, in our study, the PEDV N462A mutant exhibited reduced sensitivity to IFN-β pre-treatment, opposite to the pattern observed in most other CoVs. This underscores a distinct role of Mac1 in PEDV, potentially shaped by its enteric tropism.

Indeed, previous studies of the corresponding D445A or N462A substitutions across various other viruses consistently highlight Mac1’s role in regulating ADP-ribosylation levels in the host. For example, D10A and N24A mutations in Chikungunya virus (CHIKV) abolished hydrolysis activity and reduced viral fitness (67). Similarly, the corresponding D and N mutations in the HEV X domain suppressed RNA replication (68), and one study on rubella virus confirms these residues as catalytic or structural linchpins (69). Collectively, these studies underscore that in most + ssRNA viruses, Mac1 has important roles in regulating host immunity and its inactivation impairs viral replication or virulence. Thus, the results from our study can have a broad impact relevant to not only CoVs but also numerous other viral families.

Except for the two residues D and N examined in this study, there are other completely conserved residues in the Mac1 domain (Fig. 1B). H1045A and G1130V mutations in SARS-CoV (42) resulted in normal replication in cell culture and attenuation in mice. G1439V in the MHV-JHMV strain (corresponding to G1130V in SARS-CoV) was demonstrated to be highly attenuated compared with the MHV-N1347A mutant. The highly conserved isoleucine residue among MHV-JHM-I1440, MERS-CoV-I1238, and SARS-CoV-2-I1153 and the phenylalanine residue among MHV-JHM-F1441, MERS-CoV-F1239, and SARS-CoV-2-F1154 in the ADP-ribose binding pocket were also reported to be critical for viral virulence (45). Those residues are also conserved in PEDV (H467, I551, and F552) and are important to study in the future. Especially because the N462A mutation in the PEDV Mac1 protein did not fully eliminate its ADP-ribose binding or hydrolysis activity (Fig. 2), other substitutions or complete deletion of the Mac1 gene may be required to fully elucidate the impact of this protein on virus replication and pathogenesis. Another future research focus lies in studies of viral protein interactions. Prior work on PDCoV showed that Mac1 may act as a cofactor enhancing PLpro enzymatic activity (70). Whether this holds true in PEDV, and whether mutations like D445A or N462A impair such interactions, remains to be determined. In HEV, the Mac1’s full activity required the downstream helicase region (23, 33), suggesting that domain cooperation within viral polyproteins may be essential for full function. Comparative domain organization of nsp3 shows clear divergence between alpha-CoVs and beta-CoVs (17), indicating that interaction between Mac1 and other viral proteins may differ, supporting functional repurposing of Mac1 in PEDV, an alpha-CoV. Mapping the interaction landscape between PEDV Mac1 and other virus protein domains might represent a key future direction.

Finally, our *in vivo* data reinforce the idea that Mac1 influences *in vivo* virulence. Interestingly, the mutant virus replicated as efficiently as the wild-type virus in cell lines. This decoupling of *in vitro* replication in Vero cells from pathogenesis in pigs has implications for rational live vaccine design, because this N462A mutant can be introduced to attenuate the highly virulent virus to generate a vaccine strain but not affect the vaccine strain production *in vitro*. Besides its potential as a live attenuated vaccine development target, the PEDV Mac1 domain may also serve as a candidate for antiviral drug strategies, despite the presence of cellular macrodomains in pigs (71). Previous studies have identified compounds that specifically inhibit the ADP-ribose binding and hydrolysis activities of CoV Mac1 domains, thereby impairing viral replication (72–75). Importantly, these inhibitors showed minimal activity against alphavirus and human macrodomains *in vitro*, indicating high specificity for CoV Mac1 domains. These findings indicated that Mac1-targeting compounds could also be developed as antivirals against CoVs, provided that future studies confirm their selectivity and efficacy in the host.

Several limitations of the current study should be acknowledged. First, the mechanisms underlying the reduced IFN responses observed *in vitro* and the attenuated phenotype of icPC22A-nsp3N462A *in vivo* remain unclear. Although we attempted transcriptomic analysis of intestinal samples from Gn pigs, the sequencing data were insufficient for reliable analysis. Therefore, whether the N462A mutation similarly alters IFN responses *in vivo* remains unknown. In addition, the molecular mechanisms linking the reduced ADP-ribosyl hydrolase activity of the N462A mutant to altered host IFN responses remain to be determined. We hope this study encourages more investigation on the Mac1 domain of CoVs with different tissue tropisms.

In summary, this study reveals a previously unrecognized, potentially divergent role of the Mac1 domain in a porcine enteric alpha-CoV PEDV. Our results suggest that PEDV Mac1 induces IFN responses that are opposite to those observed in beta-CoVs. These findings raise new questions about the mechanistic diversity of CoV Mac1 and offer a novel strategy for rational design of live attenuated vaccines and antiviral development. Future work should focus on dissecting host protein targets of PEDV Mac1 and determining how these interactions shape innate immune responses in the host.

## MATERIALS AND METHODS

### Structural modeling and ligand docking

The 3D structure of the PEDV Mac1 domain (residues K431–V593) was predicted using AlphaFold2 via the ColabFold notebook (https://colab.research.google.com/github/sokrypton/ColabFold/blob/main/AlphaFold2.ipynb) with default settings (76). To visualize the interaction between Mac1 and its ligand, ADP-ribose was docked onto the predicted structure using AlphaFill (https://alphafill.eu), also with default parameters (77). Structural visualization and distance measurements were performed using UCSF ChimeraX (78).

### Cells and reagents

Vero cells (ATCC CCL-81) were cultured in Dulbecco’s modified Eagle’s medium (DMEM, Thermo Fisher Scientific, Waltham, MA, USA) in the presence of 5% fetal bovine serum (FBS, Hyclone, Logan, UT, USA) and antibiotic-antimycotic (100 U/mL penicillin, 100 μg/mL streptomycin, and 250 ng/mL amphotericin B, Thermo Fisher Scientific). LLC porcine kidney (LLC-PK1) cells (ATCC CL-101) were cultured in modified Eagle’s medium (MEM, Thermo Fisher Scientific) supplemented with 5% FBS, 1% nonessential amino acids (NEAA, Thermo Fisher Scientific), 1% antibiotic-antimycotic, and 1% HEPES (Thermo Fisher Scientific). For PEDV propagation, Vero cells were maintained in DMEM supplemented with 0.3% tryptose phosphate broth (TPB, Sigma-Aldrich, St. Louis, MO, USA), antibiotics (100 U/mL penicillin and 100 μg/mL streptomycin, Thermo Fisher Scientific), and 10 μg/mL trypsin (Thermo Fisher Scientific) as described previously (79). LLC-PK1 cells were maintained in MEM supplemented with 1% antibiotic-antimycotic, 1% NEAA, 1% HEPES, and 10 μg/mL trypsin after the viral inoculation (80, 81).

### Cloning, protein expression and purification

YFP-GAP plasmid is available from Addgene (Identify: 173080) (56). For cyan fluorescent protein (CFP)-Mac1 (wild-type, D445A mutant, and N462A mutant) constructs, gBlock genes encoding wild-type Mac1 and mutants (K431-V593) (GenBank KY499262) were ordered from Integrated DNA Technologies (IDT, Coralville, IA, USA) and then cloned into the pNIC-CFP vector (Addgene #173074). The CFP-Mac1 constructs contain an N-terminal 6× His-tag followed by CFP and a Tobacco Etch Virus (TEV) protease cleavage site upstream of the Mac1. We employed the sequence- and ligation-independent cloning (SLIC) method for cloning experiments (82). One hundred nanograms of the linearized pNIC-CFP vector was mixed with the insert in a 1:3 molar ratio and T4 polymerase for 2.5 min at room temperature, followed by 10 min on ice. The cloning reaction mixture was then transformed into *E. coli* NEB 5α competent cells. The cells were plated on LB agar plates supplemented with the selection antibiotic marker, 50 μg/mL kanamycin, and 5% sucrose as a sacB-based negative selection marker (83, 84). Plates were incubated at 37°C overnight to allow colonies to grow. The next day, the plasmid was extracted from a single colony and sequenced to confirm a successful clone.

MARylated YFP-GAP was expressed and produced as described earlier (56). The CFP-Mac1 (wild-type, D445A mutant, or N462A mutant) plasmid was transformed into *E. coli* BL21 (DE3) competent cells. The next day, several colonies were inoculated into 5 mL of Luria Broth medium containing 50 μg/mL kanamycin. The seed cultures were incubated and grown overnight at 37°C in a shaking incubator at 200 rpm. One liter of Terrific Broth autoinduction medium including trace elements (Formedium, Hunstanton, Norfolk, England, United Kingdom), containing 8 g/L glycerol and 50 μg/mL kanamycin were inoculated with 5 mL of the seed culture. Flasks were incubated at 37°C in a shaking incubator at 220 rpm until the optical density at 600 nm (OD_600_) reached 1.5. Then, the temperature was cooled down to 18°C, and cultures were incubated overnight. The cells were harvested the next day by centrifugation at 4,200 × *g* for 45 min at 4°C. The pellets were resuspended in lysis buffer containing 50 mM HEPES, pH 7.5, 500 mM NaCl, 10 mM imidazole, 10% (vol/vol) glycerol, 0.5 mM Tris(2-carboxyethyl) phosphine (TCEP), and stored at −20°C until purified.

Immobilized metal affinity chromatography (IMAC) and size-exclusion chromatography (SEC) were performed to purify proteins. Cell suspensions were thawed, supplemented with 0.1 mg/mL lysozyme, 0.02 mg/mL DNase, 0.1 mM protease inhibitor Pefabloc (Sigma-Aldrich), and incubated on ice for 20 min. The cells were kept on ice and lysed by sonication (40% amplitude for 24 cycles of 5 s on and 15 s rest). The lysate was then centrifuged at 45,000 × *g* for 45 min at 4°C to remove all clumps of cell debris or aggregated proteins. The supernatant was filtered through a 0.45 µm-pore sized filter before being loaded onto 5 mL × 2 IMAC columns charged with Ni^2+^ and pre-equilibrated with lysis buffer. The loading flow rate was kept at 2 mL/min. The column was then washed with five column volumes of lysis buffer, followed by two more washes with wash buffer containing 25 mM and then 50 mM imidazole. Proteins were eluted in an elution buffer containing 150 mM imidazole. To purify CFP-Mac1 proteins, the purification was continued with the SEC step. Eluted proteins were concentrated using 30 kDa molecular weight cut-off (MWCO) concentrator and loaded onto a Hiload 16/600 Superdex 75 (GE Healthcare, Sweden) gel filtration column, which was pre-equilibrated with SEC buffer containing 30 mM HEPES, pH 7.5, 300 mM NaCl, 10% (vol/vol) glycerol, and 0.5 mM TCEP. The purified protein was concentrated, divided into 50 μL aliquots, and flash-frozen in liquid nitrogen to be stored at −70°C. To purify Mac1 without the CFP tag, an additional step of TEV protease treatment in a 1:30 molar ratio was performed after the IMAC step. CFP tag removal was carried out overnight at 4°C during dialysis, which simultaneously removed imidazole, a known inhibitor of TEV protease. Reverse IMAC was conducted the next day to remove any impurities from the desired protein. SEC and protein storage were done as described for CFP-Mac1.

### Dissociation constant measurement

The binding affinity of PEDV Mac1 and MARylated peptide interaction was measured using quantitative FRET technology (55). A constant concentration of 500 nM CFP-Mac1 (either wild-type or mutant) was mixed with a wide range of MARylated YFP-GAP concentrations (from 50 nM to 9,000 nM) using Echo 650 acoustic liquid dispenser (Beckman Coulter Life Sciences, Indianapolis, IN, USA). The black and flat-bottom ProxiPlate-384 F microplate (Revvity, Waltham, MA, USA) was used for the assay. The sample wells were then backfilled with the FRET buffer (10 mM BTP, pH 7.0, 3% PEG 20k, 0.01% Triton X-100, and 0.5 mM TCEP) to 10 μL per well using Mantis liquid handler (Formulatrix, Bedford, MA, USA). The fluorescence emission at 477 and 527 nm (10 nm bandwidth) upon excitation at 430 nm (10 nm bandwidth) was measured. Additionally, the emission at 527 nm (10 nm bandwidth) was measured upon excitation at 477 nm (10 nm bandwidth). The measurement was performed using Spark plate reader (Tecan, Männedorf, Switzerland) (55).

### ADP-ribosyl hydrolase activity of wild-type Mac1 and mutants

We used the dot blot method to measure the hydrolysis activity of wild-type PEDV Mac1 and its mutants. MARylated SRPK2 modified by PARP10 served as the substrate for the hydrolysis activity, and nanoluciferase-fused ADP-ribosyl binder eAf1521 (Nluc-eAf1521) was employed as a luminescent probe to detect the MARylation on the blots (56). The MARylated SRPK2 substrate was prepared as described earlier (72). To test the hydrolysis activity, 10 nM of either wild-type or mutant PEDV Mac1 and 0.5 µM MARylated SRPK2 were mixed and incubated at 30°C for 2 h. After incubation time, 1 μL of the reaction solution was manually spotted onto the nitrocellulose membrane. After drying of the spots, the membrane was blocked with 5% (wt/vol) skim milk in Tris-buffered saline supplemented with 0.1% Tween-20 (TBS-T) on a shaker for 1 h at 4°C. The blocking solution was then discarded, and the membrane was incubated with 3.2 μg/mL Nluc-eAf1521 in 1% (wt/vol) skim milk solution for another hour. Then, the membrane was briefly rinsed and incubated with TBS-T on a shaker for 1 h. Finally, following a final rinsing with TBS-T, the membrane was visualized using 500 μL of 1:1,000 NanoGlo substrate (Promega, catalog number: N1110) diluted in 10 mM sodium phosphate buffer pH 7.0.

### Generation of PEDV mutant viruses using reverse genetic system

The PEDV mutants icPC22A-nsp3D445A and icPC22A-nsp3N462A were subjected to recovery based on the full-length cDNA clone of PEDV strain PC22A as described previously (57, 85). Briefly, the point mutations were introduced into the plasmid pUC19 carrying the gene fragment of icPC22A (GenBank KY499262) via NEB Q5 Site-Directed Mutagenesis Kit (NEB, Ipswich, MA, USA). Then the sequence of plasmids was confirmed by Whole Plasmid Sequencing service at Plasmidsaurus (Eugene, OR, USA) using Oxford Nanopore Technology. After the plasmids were digested by restriction enzymes, the appropriately sized cDNA inserts were purified using the QIAquick gel extraction kit (Qiagen, Hilden, Germany). All five fragments (A, B, C, D, and E) were ligated with T4 ligase (NEB) at 4°C overnight in an equal molar ratio for full-length cDNA. The ligated full-length cDNAs were purified by chloroform extraction and used as templates for *in vitro* transcription using mMessage mMachine T7 transcription kit (Ambion, Austin, CA, USA). The polyadenylated PEDV N gene transcript was generated using HiScribe T7 ARCA mRNA kit (NEB) and co-electroporated into Vero cells with the full-length transcripts at 450 V and 50 μF using a Gene Pulser II electroporator (Bio-Rad, Hercules, CA, USA). At 6–8 h after electroporation, the growth medium was discarded, and Vero cells were cultured in maintenance medium in the presence of 10 μg/mL trypsin. Cells were monitored for cytopathic effect (CPE) following infection. Once CPE was observed, the virus was harvested and designated as P0 virus. The P0 virus was then subjected to plaque assay, where five individual plaques per virus were initially picked up and propagated. The whole genomes of well-replicated ones with clear CPE were sequenced by the Linear/PCR Sequencing service at Plasmidsaurus. The clone with the correct whole genome sequence was selected for further characterization.

### Plaque assay

Monolayers of Vero cells in six-well plates were incubated with 10-fold serially diluted PEDV mutant for 1 h. After that, the inoculum was removed, and cells were washed with PBS. Then, cell monolayers were covered with overlay containing 1% agarose in MEM supplemented with 10 μg/mL trypsin and 0.3% TPB (79, 86). At 4 dpi, the cells were fixed with 10% PBS-buffered formalin for 15 min and stained with 0.2% crystal violet.

### PEDV replication kinetics

Vero, IPEC-J2, or LLC-PK1 cell monolayers in 12-well plates were infected with the corresponding viruses at an MOI of 0.01, as described previously (81, 85). After the 1-h adsorption, cell monolayers were washed with PBS (−) to remove those unbound virions and cultured in maintenance medium. Both cells and supernatants were collected at multiple time points and titrated in Vero cell monolayers in 96-well plates for 50% tissue culture infective doses (TCID_50_) by the Reed-Muench method (87).

### Interferon sensitivity assay

The sensitivity of PEDV propagation to the human IFN-β pre-treatment in Vero cells was previously validated (63). Vero cells were cultured in medium supplemented with various concentrations of human type I interferon (IFN-β) (R&D Systems, Inc., Minneapolis, MN, USA) for 18 h prior to PEDV inoculation, as previously described (81). After pre-treatment, the medium was removed, and the cells were washed three times with PBS (−) before inoculation with individual virus at an MOI of 0.01. The infected cells were incubated for an additional 24 h before harvesting supernatants and cells, which were immediately frozen at −80°C. After a single freeze-thaw cycle, the samples were centrifuged, and the supernatants were collected for viral titration by TCID_50_ assay. Interferon sensitivity was calculated as the ratio of the virus titer in IFN pre-treated cells (TCID_50_/mL) to that in untreated control cells (TCID_50_/mL).

### RNA sequencing (RNA-seq) and differential gene expression (DGE) analysis

IPEC-J2 cells were infected with individual PEDV mutants at an MOI of 3 in the presence of trypsin (10 µg/mL). At 12 hpi, supernatants were collected for titration of viral infectious titers, and cells were lysed in DNA/RNA Shield (Zymo Research, Irvine, CA, USA) and submitted to Plasmidsaurus (San Francisco, CA, USA) for RNA-seq. Quality of the fastq files was assessed using FastQC (v0.12.1). Quality-filtered reads were aligned to the reference genome (Sscrofa11.1.114) using STAR aligner (v2.7). Gene-level expression quantification was performed using featureCounts (subread package v2.1.1). Differentially expressed gene (DEG) analysis was conducted using edgeR (v4.0.16). Significant DEGs were identified by comparing cells infected with wild-type PEDV icPC22A to each individual nsp3 mutant, icPC22A vs nsp3-D445A and icPC22A vs nsp3-N462A, using an adjusted *P* value cut-off of <0.05 and an absolute log2 fold change of ≥1 (adj-*P* < 0.05 and |log2FC| ≥ 1). Functional enrichment analysis was performed using ShinyGO (v0.85.1) (88).

### Interferon induction assay

LLC-PK1 cells were inoculated with individual PEDV mutants at an MOI of 1. After 1 h of adsorption, the virus inoculum was removed, and the cells were washed with PBS (−). At 12 hpi, the supernatants were collected to titrate viral infectious titer by TCID_50_, and the total cellular RNA was extracted with TRIzol reagent (Thermo Fisher Scientific). Then, cellular DNA was removed by treating with DNase I (Qiagen). Viral total N gene RNA levels were determined by PEDV-specific reverse transcription-quantitative PCR (RT-qPCR) using the OneStep RT-PCR kit (Qiagen) with forward and reverse primers (forward, 5′-CGCAAAGACTGAACCCACTAAC-3′; reverse, 5′-TTGCCTCTGTTGTTACTTGGAGAT-3′) and a probe (5′-FAM-TGYYACCAYYACCACGACTCCTGC-BHQ-3′) (85). The copy number was determined by standard curves. One microgram of total RNA was reverse transcribed using SuperScript IV Reverse Transcriptase and random hexamers (Thermo Fisher Scientific). The cDNA was subjected to quantitative PCR using SYBR green PCR mix (Thermo Fisher Scientific) according to the manufacturer’s instructions. Primers for IFN detection were described previously (89). The β-actin gene was used as an internal control. The threshold cycle (CT) values for target genes and the differences in their CT values (ΔCT) were determined. Relative transcription levels of target genes are presented as fold changes relative to the mock controls using the 2^−ΔΔCT^ threshold method (90).

### Gnotobiotic piglet infections

Gn pigs were delivered from PEDV-free sows via C-section, as described previously (91). Three to four Gn piglets were housed in one isolator. At 3 days of age, Gn piglets were orally inoculated with 100 TCID_50_/pig of icPC22A-nsp3N462A (*n* = 5), icPC22A (*n* = 6, as positive control), or MEM (*n* = 4, as mock). In our lab, we titrate PEDV using plaque assay for plaque-forming units (PFU) and/or microwell assay for TCID_50_, and the titers are similar using these two methods for PEDV icPC22A. So, this inoculation dose of PEDV corresponds to a value of 100–1,000 times the 50% porcine diarrhea dose (PDD_50_) of the icPC22A strain in 4-day-old cesarean-derived, colostrum-deprived piglets (57). One pig at the acute infection phase from each of the icPC22A-nsp3N462A and icPC22A groups was euthanized for histopathological examinations. After virus inoculation, clinical signs, including diarrhea and vomiting, were monitored daily. Rectal swabs were collected daily. The severity of diarrhea was scored based on the fecal consistency (FC) in individual pigs: 0, solid; 1, pasty; 2, semiliquid (moderate diarrhea); and 3, liquid (severe diarrhea) (85). Viral RNA in the rectal swab samples was extracted using the MagMax RNA isolation kit (Thermo Fisher Scientific) and MagMAX Express instrument (Thermo Fisher Scientific) according to the manufacturer’s instructions, and viral RNA shedding titers were determined targeting the N gene as previously described (85). The copy number was determined by standard curves.

### Immunohistochemical staining

The formalin-fixed jejunum and ileum samples were processed using a non-biotin polymerized horseradish peroxidase system (BioGenex Laboratories, San Ramon, CA, USA) for IHC staining. Tissues were counterstained with hematoxylin. Monoclonal antibody SD17-103 (gift of Eric Nelson and Steven Lawson, South Dakota State University) targeting PEDV N proteins was used as the primary antibody. Ratios of villous height-to-crypt depth (VH:CD) of each group were measured as described before (85). For each intestinal section, 15 villi and crypts were selected and measured from different sections.

### Statistical analysis

The statistical analyses were performed using GraphPad Prism 10.3. Data in Fig. 2, 3C, 4, 5, and 7B are shown as mean ± standard deviation. Figures 2D, 3D, 4, 5, and 7B were analyzed by one-way analysis of variance (ANOVA), followed by Tukey’s multiple-comparison test. Survival differences in Fig. 6C were analyzed using the Log-rank (Mantel-Cox) test. Groups with significant differences (*P* < 0.05) were indicated with different letters or asterisks (*, *P* < 0.05; **, *P* < 0.01; ***, *P* < 0.005; and ****, *P* < 0.001).

## Data Availability

The data supporting the findings of this study are available within this article and its supplemental material. The raw RNA-sequencing data generated in this study have been deposited in the NCBI Sequence Read Archive (SRA) under BioProject accession number PRJNA1510013.

## SUPPLEMENTAL MATERIAL

Table S1 jvi.01251-26-s0001.docx DEGs that are associated with ADP-ribosylation-related pathways.

ASM does not own the copyrights to Supplemental Material that may be linked to, or accessed through, an article. The authors have granted ASM a non-exclusive, world-wide license to publish the Supplemental Material files. Please contact the corresponding author directly for reuse.
