# Supplementary material for: Mutations in the macrodomain 1 of nsp3 attenuate PEDV virulence in vivo without impairing in vitro replication
Source: J Virol. 2026 Sep 9;100(10):e01251-26. doi: 10.1128/jvi.01251-26 (PMC13712927; doi:10.1128/jvi.01251-26)
Supplement: Supplementary file 1 [file jvi.01251-26-s0001.docx]

| **Supplementary Table 1.** **DEGs that are associated with ADP-ribosylation-related pathways.** | | | |
| --- | --- | --- | --- |
| Gene | Annotation | Functions (from GeneCards) | Relevance with ADP ribosylation or coronavirus infection |
| TXNIP | Thioredoxin-interacting protein | - Encodes a thioredoxin binding protein - Thioredoxin is a thiol-oxidoreductase that is a major regulator of cellular redox signaling which protects cells from oxidative stress. - This protein may act as an oxidative stress mediator by inhibiting thioredoxin activity or by limiting its bioavailability - Transcriptional repressor - Inhibits proteasomal degradation of DDIT4, thereby contributes to the inhibition of the mTORC1 - Upregulated and activated by ROS and oxidative stress (<https://bpb-us-e2.wpmucdn.com/sites.wustl.edu/dist/f/1131/files/2025/08/TXNIP_AJP-2025.pdf>) | Interacts with PARP1 in primary macrophages  <https://pubmed.ncbi.nlm.nih.gov/35098371/>   - PARP-1 was also a bridge between NLRP3 and TXNIP and participated in NLRP3/TXNIP complex formation for inflammasome activation. - Overall, PARP-1 positively regulates NLRP3 inflammasome activation via increasing ROS production and interaction with TXNIP and NLRP3, leading to PARylation of NLRP3   PARP-1 regulates TXNIP localization and function in activating plasma membrane signaling and cell survival  <https://pmc.ncbi.nlm.nih.gov/articles/PMC3876410/>  PEDV also triggers NLRP3 inflammasome (in IPEC-J2)  <https://pmc.ncbi.nlm.nih.gov/articles/PMC11680147/> |
| ENSSSCG00000002520 | Solute carrier family 25 member 29 (SLC25A29) | Mitochondrial transporter/mitochondrial carnitine-acylcarnitine-like translocase/Ornithine (ORNT3) translocase   - Recent studies showed that it transports basic amino acids (with a preference of arginine and lysine) | n/a There’s another member of SLC25 family (SLC25A51) that helps transport NAD+ into the mitochondrial matrix. Its absence increases nuclear NAD+ which enhances PARP1-mediated ADP ribosylation  <https://www.nature.com/articles/s41467-020-19871-x>  <https://pmc.ncbi.nlm.nih.gov/articles/PMC10516648/> |
| ENSSSCG00000056294 | Looks like it’s IL28B (aka Interferon Lambda 3; IFNL3) | Cytokine induced by viral infection   - Plays a critical role in the antiviral host defense, predominantly in the epithelial tissues   - Demonstrates the greatest antiviral potency against hepatitis C virus (HCV) in vitro (https://www.gastrojournal.org/article/S0016-5085(14)01332-8/fulltext) - Acts as a ligand for the heterodimeric class II cytokine receptor composed of IL10RB and IFNLR1, and receptor engagement leads to the activation of the JAK/STAT signaling pathway resulting in the expression of IFN-stimulated genes (ISG), which mediate the antiviral state. - Plays a significant role in the antiviral immune defense in the intestinal epithelium - Exerts an immunomodulatory effect by up-regulating MHC class I antigen expression. | In COVID-19, reduced IFN-λs expression has been proven to be related with severe disease and worse prognosis (<https://pubmed.ncbi.nlm.nih.gov/34492226/>)  The 3’ UTR of the IFNL3 mRNA interacts with ZAP  <https://pmc.ncbi.nlm.nih.gov/articles/PMC7240801/>   - Zinc antiviral protein (aka PARP13; is an RNA binding protein) - this protein is considered antiviral restriction factor against several classes of viruses (Alphaviruses, Filoviruses, Retroviruses) |
| CCL4 | C-C Motif Chemokine Ligand 4 | - A mitogen-inducible monokine that binds to CCR5 (chemokine receptor) - Secreted and has chemokinetic and inflammatory functions - One of the major HIV-suppressive factors produced by CD8+ T-cells | - Was shown to be significantly upregulated in cells infected with swine enteric CoVs   <https://pmc.ncbi.nlm.nih.gov/articles/PMC8810500/>   - Linked to PARP-1 (PARP-1 mediated gene expression modulation in activating T ells)   - Expression of several Th1 chemokines such as Xcl1**, Ccl4** and Ccl9 seem to be also increased in Parp-1^-/-^ T cells in response to anti-CD3 + anti-CD28 activation   <https://pmc.ncbi.nlm.nih.gov/articles/PMC2375913/> |
| IL29 | Aka IFNL1 (Interferon Lambda 1)  Closely related to IL28B and IL28A | Functions like IL28B | ADP ribosylation regulated the signaling function of IFN-lambda   - ADP-ribosylation inhibits the signaling functions of IFN-γ <https://www.frontiersin.org/journals/immunology/articles/10.3389/fimmu.2021.642545/full> |
| APOBEC1 | Apolipoprotein B MRNA Editing Enzyme Catalytic Subunit 1 | Cytidine deaminase enzyme   - catalyzes the cytidine to uridine in both DNA and RNA - post-transcriptional editing of a variety of mRNAs - Antiviral restriction factor against HIV-1, Herpes simplex virus-1   <https://journals.asm.org/doi/10.1128/jvi.05288-11>  <https://pmc.ncbi.nlm.nih.gov/articles/PMC2588513/> | Can edit C to U in SARS-CoV-2 RNA  <https://pmc.ncbi.nlm.nih.gov/articles/PMC9470679/>  (plays a role in modifying viral genomic RNA structure)  The editing capability can mutate DNA 🡪 DNA damage, which could activate DNA repair mechanism mediated by PARP-1 and ADP ribosylation (<https://pmc.ncbi.nlm.nih.gov/articles/PMC5322727/>) |
| COL1A1 | Collagen Type I Alpha 1 Chain | Member of group I collagen | In COVID-19, this gene has been found to increase expression in lung tissue and airway macrophage (induces pulmonary fibrosis and associates with poor prognosis)  <https://pmc.ncbi.nlm.nih.gov/articles/PMC10648646/>  downregulated in tumor treated with PARP-1 inhibitor (DPQ)  <https://aacrjournals.org/cancerres/article/66/11/5744/525712/Inhibition-of-Poly-ADP-Ribose-Polymerase-Modulates> |
